# Teaching computational toxicology as new approach methodologies in the era of next-generation risk assessment

**DOI:** 10.3389/ftox.2026.1825356

**Published:** 2026-10-14

**Authors:** Shengnan Liu, Jin Li, Dawei Tang, Kan Shao, Qiao Xue, Paul L. Carmichael, Jingbo Pi, Qiang Zhang

**Affiliations:** 1 Key Laboratory of Environmental Stress and Chronic Disease Control and Prevention Ministry of Education, China Medical University, Shenyang, China; 2 Program of Environmental Toxicology, School of Public Health, China Medical University, Shenyang, China; 3 Safety, Environmental and Regulatory Science, Unilever, Colworth Science Park, Sharnbrook, United Kingdom; 4 Department of Environmental and Occupational Health, School of Public Health, Indiana University Bloomington, Bloomington, IN, United States; 5 College of Resources and Environment, University of Chinese Academy of Sciences, Beijing, China; 6 Gangarosa Department of Environmental Health, Rollins School of Public Health, Emory University, Atlanta, GA, United States

**Keywords:** computational toxicology, NAMs, NGRA, risk assessment, teaching

## Abstract

The global initiative toward non-animal chemical testing methods has recently gained significant momentum, requiring developing new approach methodologies (NAMs) as transformative solutions for next-generation risk assessment (NGRA). NAMs refer to any *in chemico, in silico*, and *in vitro* technology, methodology, or combination that do not rely on animals. A range of computational toxicology methods are becoming critical components within the NGRA framework across multiple testing tiers. These approaches are needed to support hazard characterization and prioritization for further testing, calculate points of departure, identify modes of action, and bridge the data gaps between *in vitro* assays and *in vivo* exposures as well as apical health endpoints. The core technologies of computational toxicology include (i) quantitative structure activity relationship, (ii) benchmark dose modeling, (iii) bioinformatic analysis of omics data, (iv) physiologically based kinetic modeling, (v) quantitative adverse outcome pathway modeling, (vi) molecular docking and dynamics, and (vii) more recently, machine learning and artificial intelligence tools. The NAM community, in academia, industry, and regulatory agencies, are facing increasing demand for training current and future professionals to master these skills. In this article, we described our perspective, experiences, and challenges in disseminating these computational toxicology techniques. Unlike established disciplines, the curriculum of computational toxicology and associated software tools are constantly evolving, requiring instructors to adapt in a timely manner. Challenges abound, we are embraced with an unprecedented opportunity that educating next-generation toxicologists is a critical effort of this revolutionary movement of chemical toxicity testing and safety assessment.

## 1 Background

New approach methodologies (NAMs) have emerged as a potential transformative solution for next-generation risk assessment (NGRA) of exposures to environmental pollutants, consumer products, and drugs (Baltazar et al., 2020; Schmeisser et al., 2023; Sewell et al., 2024; Deepika et al., 2025; Herron et al., 2025; Leso et al., 2025; Sheng et al., 2025; Kim and Choi, 2026; Liu et al., 2026). The NAM-based NGRA emphasizes an exposure-led, hypothesis-driven approach to ensure that regulatory decisions are human health protective (Han, 2023; Grimm, 2024; Zuang et al., 2024; Baltazar et al., 2025; Mehta et al., 2025; Paul Friedman et al., 2025). It follows a tiered decision-making process (Berggren et al., 2017; Thomas et al., 2019; Cable et al., 2025). Tier 0 utilizes existing data/information and *in silico* tools, Tier 1 generates new human cell-based *in vitro* data, and Tier 2 performs refined, targeted assays to further reduce uncertainty (Cable et al., 2025). A variety of computational toxicology techniques are required as *in silico* NAM tools to bridge the data gap in the tiered process (Rusyn and Daston, 2010; Reisfeld and Mayeno, 2012; Zhang et al., 2018; Cable et al., 2025), including (i) quantitative structure activity relationship (QSAR) modeling, (ii) benchmark dose (BMD) modeling, (iii) bioinformatics, (iv) physiologically based kinetic (PBK) modeling, (v) quantitative adverse outcome pathway (qAOP) modeling, (vi) molecular docking/dynamics, (vii) machine learning (ML) and artificial intelligence (AI) (Table 1).

**TABLE 1:** Computational toxicology techniques in tiered NAMs.

Technique	Description	Applicable tiers	Software	Prerequisites
QSAR	Statistical or ML/AI methods to construct quantitative mapping between the molecular structure of a chemical and its physiochemical/biological properties	0, 1	VEGA ADMET-AI	Organic chemistry; general knowledge in statistical regression and ML
BMD	Statistical curve-fitting methods that calculate the PoD dose/concentration for a predefined response level with uncertainty considerations	1	BMDS (bmdsonline.epa.gov), BBMD (benchmarkdose.com), EFSA-BMD (r4eu.efsa.europa.eu), PROAST (proastweb.rivm.nl), and ToxicR	Statistical modeling
Bioinformatics for omics	Statistical tools for omic data to identify gene- and pathway-level BMD and spatial/cell type-specific responses	1	BMDExpess, BBMD, and FastBMD	Statistics; KEGG and GO
PBK	Numerical integration methods that mechanistically simulate the ADME of chemicals in humans and animals	1, 2	R, Python, Julia, COPASI, Berkeley Madonna, PK-Sim, and *httk* in R	ADME concepts; Mammalian gross anatomy; Calculus and numerical integration
qAOP	Numerical integration methods that simulate MIEs and KEs in AOPs	2	R, Python, Julia, COPASI, MOBI, and Berkeley Madonna	Molecular and cell biology; Calculus and numerical integration
Molecular docking/dynamics	Numerical methods that mechanistically simulate the binding interactions between two molecules	0, 2	Autodock, Autodock Vina, Surflex, SwissDock, rDock, GROMACS and Amber	Molecular mechanics and thermodynamics
ML/AI	Computational methods that can predict protein structure, docking, toxicity, and PK/PD parameters, and perform public data collection and curation, systematic review, and model construction etc	0, 1, 2	Deep docking, AlphaFold, Boltz-2, Sysrev, DeepCodex AllerCatPro, Claude, ChatGPT, Gemini, and DeepSeek	Statistics; traditional ML methods and neural networks

Integrating computational toxicology into higher education curriculum is essential to prepare the next-generation scientists for the emerging opportunity and challenge in toxicity testing (Hall and Johnson, 2025; Payton et al., 2025). Computational toxicology involves cross-disciplinary knowledge and skills in chemistry, biology, physiology, toxicology, mathematics, programing, and engineering (Reisfeld and Mayeno, 2012). Some of the techniques, such as QSAR, BMD, and PBK modeling, have been traditionally taught as stand-alone courses in academic settings or workshops. However, rarely are they taught collectively as a suite of *in silico* NAM tools. There are limited ongoing efforts that systemically promote computational toxicology education in the context of NGRA (AFSA, 2025; Payton et al., 2025). In the past 10 years from 2015 to 2025, the School of Public Health at China Medical University (SPH-CMU) has hosted six national computational toxicology workshops (CTW) by collaborating with world-class scientists specialized in NAMs from Unilever, Emory University, Indiana University, The Hamner Institutes, US EPA, and institutions in China. These workshops include (i) “Computational Systems Biology and Dose Response Modeling” (CTW, 2015), (ii) “PBPK/PBTK Modeling” (CTW, 2017; CTW, 2023), (iii) “qAOP Modeling” (CTW, 2021), (iv) “21st Century Toxicity Testing and Computational Toxicology” (CTW, 2019), and (v) “Computational Toxicology for NAM in the Era of NGRA” (CTW, 2025). Offered as free, 3- or 4-day short courses in the format of lectures and hands-on computer exercises, these workshops were attended collectively by about 660 trainees from over 60 universities and research institutes in China. In late 2023 we conducted an informal survey among the 360 attendees of the 2019-2023 workshops through an online platform, WenJuanXing (www.wjx.cn, survey questions and results available upon request). Among the 60 who responded, 67% claimed that they had begun applying NAMs in their research, 12% had published NAMs related papers, 10% had taught NAMs, 15% had mentored students in NAMs, 8.33% had secured NAMs-related funding, 37% had significantly reduced the use of animals, and 25% had stopped using animals in toxicity testing practice. Being recognized for the impact the series of workshops have made, the SPH-CMU was awarded the 2024 Lush Training Prize.

These workshops have served to motivate, support, and lead young toxicologists to embrace NAMs early in their career of chemical safety and risk assessment. We envisage that as many of these young trainees become professional elites in the coming years, with some moving into influential positions, their earlier exposure to and adoption of computational toxicology will help shape the chemical toxicity testing landscape in the direction of NAMs. As instructors, we have learned a great deal through these workshops. Here, we would like to share our experience and perspective on teaching computational toxicology techniques as *in silico* NAM tools.

## 2 Teaching computational toxicology as NAM tools

### 2.1 General philosophy

Students engaged in computational toxicology training often come from diverse backgrounds, including biology, chemistry, toxicology, preventive medicine, basic medical science, environmental health sciences and engineering. The vast majority often lack quantitative skills. The instructors must keep in mind that they are facing an audience who are likely to experience mental challenges upon seeing mathematical equations and programming scripts. The instructors must be prepared to break down the equations and lines of scripts into basic components and explain to the students at great lengths, at least at the beginning of the teaching. It is always helpful to use examples students are familiar with to provide some context while explaining complex concepts and algorithms. The teaching should adhere to a format of lectures followed by hands-on computer exercises. These exercises are extremely critical as they will help strengthen the understanding of the concepts and theories in an interactive way. The software used in the teaching should follow the principles of freeware, open sources, and user friendliness. Coherent case studies should be used to demonstrate how these techniques are applied in real-world NAM practice and their limitations.

### 2.2 Individual computational toxicology techniques

#### 2.2.1 QSAR modeling

QSAR is a mathematical modeling technique that uses statistical or ML methods to construct quantitative mapping between a chemical’s structure and its physiochemical/biological properties (Combes, 2012; Muratov et al., 2020). Capable of screening many chemicals, QSAR modeling has become an indispensable Tier 0/1 tool for NAM-based risk assessment (Huang et al., 2021; Cable et al., 2025; Evangelista and Papa 2025; Sheng et al., 2025).

A full grasp of QSAR modeling skills, especially learning how to build a model from scratch, requires a dedicated semester-long course with heavy coding and knowledge in organic chemistry, statistical regression, and ML. For short-term training or as a module in a course, the focus should be on its applications and helping students to achieve the following competencies: (i) understand algorithmic principle of QSAR, (ii) aware of well-established QSAR tools (Table 1), (iii) acquire skills to use at least one tool, (iv) aware of their applicable domains and limitations.

For QSAR tools like VEGA and ADMET-AI (Shamsara, 2017; Benfenati et al., 2019; Bachorz et al., 2024; Swanson et al., 2024), the operation is as simple as providing the SMILES structure of the inquiry chemical then receiving predicted results. Hands-on exercises can focus on predicting biological, toxicological and physicochemical endpoints relevant to the case studies. The limitation and applicability domain of these tools should be communicated to prevent overuse and abuse because QSAR models tend to be biased toward the categories of chemicals used for model development. Without properly evaluating structural similarity, descriptor ranges, and mechanistic boundaries to decide if a chemical falls within the model’s reliable prediction space, false positive and negative predictions can arise.

#### 2.2.2 BMD modeling

The chemical concentration or dose that produces a sufficient deviation from the basal functional state, which would have resulted in an adverse outcome, is defined as point of departure (PoD) (Paul Friedman et al., 2020; Lu et al., 2025). In the last 2 decades, the BMD method, originally proposed by Kenneth S. Crump, has become the mainstream approach to determine PoD (Crump, 1984). BMD modeling is a statistical curve-fitting process that uses a finite set of predefined mathematical functions to estimate the concentration/dose of a chemical associated with a predetermined benchmark response (BMR), with considerations of data uncertainty.

Teaching BMD modeling in a workshop setting can focus on the following competencies: (i) basic principle and historical context, (ii) aware of existing BMD tools (Table 1), (iii) learn to use at least one BMD tool, (iv) learn how to select the best model(s), (v) aware of the tool’s limitation. These competencies can be achieved via the following modules: (i) Historical perspective on EC50, NOEAL/LOEAL, and BMD; (ii) Common functions used for different data types (continuous, dichotomous, and categorical); (iii) The frequentist vs. Bayesian approach; (iv) Available BMD software tools and their pros and cons; (v) Exercises by using one BMD tool. These BMD tools have been implemented in various software packages or web-based applications, including BMD Software (BMDS) developed by the US EPA, the Bayesian BMD modeling (BBMD) system initiated in Dr. Kan Shao’s lab and developed by DREAM Tech, LLC (Shao and Shapiro, 2018), the European Food Safety Authority (EFSA) BMD tool (Verlinden and Machteld, 2024), the Probabilistic Risk Assessment Software Tool (PROAST) software developed by the Holand Rijksinstituut voor Volksgezondheid en Milieu (EFSA, 2011), and ToxicR as a R-package (Wheeler et al., 2023). During exercises using one of these tools, students should learn how to properly evaluate the quality of the fitted curves and make sound judgement on which fitted curve(s) should be used to derive the lower limit of the one-sided 95% confidence interval for BMD, i.e., BMDL, as the PoD.

#### 2.2.3 Bioinformatic analysis of omics data

Transcriptomics has emerged as the most useful omic tool due to its relatively low cost and established consensus that transcriptomic PoDs (tPoDs) are generally within one order of magnitude of the PoDs of associated apical endpoints in animal studies (Thomas et al., 2013; Thomas et al., 2019). *In vitro* transcriptomic concentration-response assays have become a must-have component in NAM-based NGRA, complementing targeted assays (Thomas et al., 2019). The adoption of other omics such as proteomics and phospho-proteomics is also emerging with their roles in defining PoDs at early time and at lower doses being increasingly appreciated (Zhang et al., 2022; Chen et al., 2023; Li et al., 2023).

The teaching should focus on transcriptomic BMD using RNA-sequencing data, aiming on the following competencies: (i) understanding how genes belonging to different functional pathways, including Gene Ontology (GO), Kyoto Encyclopedia of Genes and Genomes (KEGG), and Reactome, are grouped together and how pathway-level tPoDs are determined based on the relative weights and enrichment of these genes, (ii) aware of existing transcriptomic BMD tools, their pros and cons, and the transcriptomic data type and format, and (iii) use one of these tools to calculate gene- and pathway-level tPoDs. Existing tools include (i) BMDExpress developed through collaborations between the NTP of NIEHS, Health Canada, USEPA, and Sciome LLC. (Phillips et al., 2019), (ii) the *BMD Analysis for Genomic Data* module on the BBMD platform (Ji et al., 2022), and (iii) FastBMD, a free web-based tool supporting pathway-level tPoD calculation for 13 species (Ewald et al., 2021).

#### 2.2.4 PBK modeling

PBK modeling is a technique that mechanistically simulates the absorption, distribution, metabolism, and excretion (ADME) of chemicals in an organism such as humans (Upton et al., 2016). A system of coupled ordinary differential equations (ODEs) is formulated to describe the rates of change of the amounts of the chemical in blood-connected tissue compartments. PBK models are increasingly used as an indispensable *in silico* *in vitro* to *in vivo* extrapolation (IVIVE) NAM tool to predict the external exposure level that would give rise to a specific tissue PoD concentration of the chemical obtained from *in vitro* assays (Yu et al., 2020; Zhang et al., 2020; Cable et al., 2025).

The teaching should focus on the following competencies: (i) understand mechanistic principles of chemical ADME, (ii) determine model structures, (iii) write and parameterize ODEs by using one simulation tool, (iii) fit model to data, and (iv) perform sensitivity analysis. A typical PBK modeling course can include the following modules: (i) Historical background; (ii) Basic concepts including partition coefficient, plasma fraction unbound, tissue distribution (flow-limited, diffusion-limited, and transporter-limited); (iii) Flow-limited and biochemically limited clearance; (iv) Different exposure routes, such as oral, inhalation, and dermal; (v) Full model for both acute and chronic exposure scenarios; (vi) Parameterization methods and resources; (vii) Sensitivity analysis, variability and uncertainty, and population PK.

In our experiences, students do not need to have a strong math background to understand the basic ODEs as they are simple algebraic expressions on the right side of the equations and are solved numerically. It is important to convey that how chemical-specific parameters, especially those sensitive ones (for example, liver clearance, fraction unbound in plasma, etc.), are obtained–by either QSAR-based prediction, *in vitro* measurement, or human data-based estimation–determines the uncertainty levels thus the confidence in the PBK model (Middleton et al., 2022; Cable et al., 2025). A PBK model can be built and simulated in almost any general programming languages equipped with an ODE solver package, such as R, Python, and Julia (Apraun, 2025). However, for programming novices, learning how to code may get in the way of learning PBK modeling itself. Our experience suggests that a dedicated ODE solving program that has a graphic interface, such as Berkeley Madonna (Marcoline et al., 2022), COPASI (Hoops et al., 2006), and PK-Sim (Lippert et al., 2019), can be an effective teaching tool, which insulates students from the error-prone coding chores. Students can also be introduced to pre-built PBK tools such as the *httk* package in R as a resource that can make quick tissue dosimetry predictions for thousands of compounds (Pearce et al., 2017).

#### 2.2.5 qAOP modeling

Based on the AOP framework, an apical health endpoint is the result of whole-body response to alterations in one or multiple interconnected organs and tissues. qAOP models that can capture such complex organ-organ interactions to achieve quantitatively accurate predictions for *in vivo* PoDs are needed as *in silico* refinement tools in Tier 2 of NGRA. The most commonly approach for qAOP modeling is deterministic or stochastic numerical simulations (Jeong et al., 2025). It requires a computational systems biology approach that mathematically captures the molecular and cellular key events (KE) across scales in the form of ODEs (Conolly et al., 2017; Zhang et al., 2018; Perkins et al., 2019; Di Tillio and Beltman, 2024). Multiple KEs further form networks, generating complex dynamic behaviors, including multistability, homeostasis, and oscillation, and as a result, nonlinear dose responses.

A qAOP modeling course can aim to achieve the following competencies: (i) principles of mass-action kinetics; (ii) dynamic properties of positive, negative feedback, and feedforward; (iii) stability and bifurcation analysis; and (iv) deterministic vs. stochastic simulations. The course can be taught with the following modules: (i) Simple processes including synthesis, degradation, reversible binding, and Michaelis-Menten kinetics; (ii) Signal amplification and ultrasensitivity; (iii) Positive feedback, double negative feedback, bistability, and switch-like responses; (iv) Negative feedback, adaptation, and homeostasis; (v) Negative feedback and biological oscillation; (vi) Feedforward, noise filtering, signaling delay, and hormesis; (vii) Complex biological circuits such as oxidative stress response and cell cycle regulation.

Teaching qAOP modeling is generally more intellectually challenging than teaching PBK modeling, involving a series of mathematical concepts in nonlinear dynamical systems, including Jacobian matrix, eigenvalues/eigenvectors, fixed point, saddle-node bifurcation, Hopf bifurcation, and limit cycle. Our experience is to teach these concepts in a biochemical context students are familiar with. qAOP models can be built and simulated in almost any general programming languages as well as Berkeley Madonna, COPASI, and MOBI (Lippert et al., 2019).

#### 2.2.6 Molecular docking/dynamics

Molecular docking is a computational technique traditionally used to predict the binding interactions between a small molecule (ligand) and a macromolecular target (Morris and Lim-Wilby, 2008). It can be used in NAM to predict molecular initiating events (MIEs), prioritize compounds for testing, and assess potential off-target toxicity (Cozzini and Agosta, 2025). Docking algorithms incorporate molecular mechanics, thermodynamics, and sometimes solvent effects; it requires the 3D structure of the macromolecule (Jumper et al., 2021; Abramson et al., 2024), and knowledge of the potential binding pockets.

Molecular dynamics (MD) simulates the physical movements of atoms and molecules over time by solving Newton’s equations of motion, providing a dynamic and high-resolution view of toxicant-target interactions (Xue et al., 2019; Liu et al., 2024). The applications of MD in NAM include: (i) Validation of MIEs: refining and validating the stability of toxicant-receptor complex docking poses; (ii) Conformational toxicity: investigating target flexibility, allosteric modulation, and identification of binding pockets not apparent in static structures; (iii) Complex environmental interactions: using multi-scale or coarse-grain simulations to model the permeation of emerging pollutants (such as PFAS) across lipid bilayers, providing insights into bioaccumulation and cellular uptake mechanisms (Kuo et al., 2017).

The competencies of learning molecular docking/dynamics should include (i) the mechanistic concepts behind these simulations; (ii) limitations such as protein flexibility, scoring accuracy, and false positive; (iii) master at least one software platforms (Table 1). For MD, it can be challenging for students to transition from static “snapshots” of chemical binding to a dynamic, temporally resolved biological reality. Although MD requires a steeper learning curve and higher computational cost, teaching students how to balance these factors against the need for rigorous predictive accuracy is essential.

#### 2.2.7 ML/AI application in NAM

ML/AI has revolutionized fields such as protein folding, ligand-receptor binding, and activity prediction, offering powerful *in silico* NAM tools for drug development and chemical risk assessment (Singh et al., 2024). Neural network-based ML models have tremendously improved the accuracy of QSAR models to predict liver toxicity, endocrine disruptors, neurotoxicity, ADME parameters, allergenicity, transcriptomic profiles, and uncertainty of predicted bioactivity, etc. (Donner et al., 2018; Gini et al., 2019; Jiang et al., 2020; Allen et al., 2022; Collins and Barton-Maclaren, 2022; Nguyen et al., 2022; Khan et al., 2024; Chou et al., 2023; Zhang et al., 2025). Recently, PK/PD models based on Neural-ODE structures have been developed to predict different dosing scenarios for clinical drugs (Chen et al., 2018; Lu et al., 2021a; Lu et al., 2021b). AI tools have been developed to predict protein folding, including Deep Docking (Gentile et al., 2020), AlphaFold/AlphaFold2/AlphaFold3 (Jumper et al., 2021; Abramson et al., 2024). Furthermore, natural language processing has been utilized to develop online tools such as Sysrev for data curation and systematic evidence review, with many applications in toxicology including developing AOP structures (Bozada et al., 2021; van Ertvelde et al., 2023). As the field is rapidly evolving, how to incorporate emerging, relevant ML/AI tools poses a crucial challenges in future NAM training. The competencies should include (i) keep students abreast of the various capabilities of ML/AI tools in NAM; (ii) learn several well-established tools, such as deep network-based QSAR models; (iii) aware of their limitations.

## 3 Conclusion

With the increasing adoption of NAMs in chemical risk assessment and drug development, a range of traditional and emerging computational toxicology methodologies are being advanced and applied in regulatory initiatives and guidelines. These *in silico* tools are essential for effective implementation of NAMs within the NGRA framework and modern safety sciences. Although teaching and training on these interdisciplinary subjects present challenges, it is imperative to prepare future toxicologists, risk assessors, and regulatory professionals with the competencies necessary to advance NAM in chemical testing.

## Data Availability

The original contributions presented in the study are included in the article/supplementary material, further inquiries can be directed to the corresponding authors.
